# Preparation of Double-Networked Slow-Expanding Nanomicrospheres and Evaluation of Drive Modulation Performance

**DOI:** 10.3390/molecules29225378

**Published:** 2024-11-14

**Authors:** Qiaolin Zuo, Zhenzhong Fan, Qingwang Liu, Yuanfeng Fu, Luoqi Cui, Junfeng Yang

**Affiliations:** 1 Key Laboratory of Enhanced Oil Recovery, Northeast Petroleum University, Ministry of Education, Daqing 163318, China; zuoqiao0906@163.com (Q.Z.); 18953328731@163.com (L.C.); 17205229039@163.com (J.Y.); 2 Bohai Rim Energy Research Institute, Northeast Petroleum University, Qinhuangdao 066004, China; lliuqingwang@163.com

**Keywords:** reverse microemulsion method, nanomicrospheres, oil field regulation and flooding, enhanced oil recovery, slow expansion performance, composite network

## Abstract

Aiming at the problem of excessive swelling of conventional microspheres for oilfield use, a novel amphiphilic polymerizable crosslinker (AE) was synthesized by quaternary ammonium modification of an unstable crosslinker (AE) using acrylamide, 2-acrylamido-2-methylpropanesulfonic acid as the monomers, N,N′-methylene bisacrylamide as the stabilizing crosslinker, ammonium peroxysulfate and sodium bisulfite as the initiator, and water as the solvent by using a reversed microemulsion method. Double-networked nanomicrospheres were prepared. The preparation conditions of the microspheres were optimized by the surface response method, focusing on the effects of the initiator addition and reaction temperature, and total crosslinker addition on the formation of nanomicrospheres. The samples were characterized by FTIR, TGA, laser particle sizer, and SEM to evaluate the retarded expansion performance and the modulation drive performance. The results showed that the optimal conditions for the preparation of microspheres were m(oil phase):m(water phase) = 3:2, stirring speed of 550 r/min, total crosslinking agent dosage of 0.6% (based on the total mass of monomers, hereinafter the same), initiator dosage of 0.30%, reaction temperature of 45 °C, and reaction time of 4 h. Compared with the conventional polymer microsphere PAM, PAE was slow-expanded for 45 d at 60 °C, and the expansion multiplier was about 16 times, with slow-expansion characteristics; the blocking rate of PAE reached 98.3%, the oil repulsion rate was 73.11%, and the increase in the recovery rate could be up to 11.23%. In this paper, a new type of nanomicrosphere material is investigated to realize the efficient implementation of oil field conditioning and driving.

## 1. Introduction

During oil production, excessive water production may occur due to the diversion fracture [1], reservoir layer, and high permeable layer [2,3], problems that may negatively affect the recovery rate in the production process. In the high-humidity stage, it is easier for the injected fractional fluid [4] to flood high-permeability layers or flow along waterways, resulting in low flooding efficiency [5]. Typically, up to 70% of the residual oil remains in the low-permeability layer, which not only affects the final recovery rate, but also significantly reduces the profitability of oil and gas production. In addition, large amounts of produced water [6] increase the pumping costs and oil–water separation costs.

Historically, many recovery methods, including polymer flooding [7], foam flooding [8], and gel adhesive plugging [9], have been widely used worldwide to control excess produced water and improve oil recovery. In recent years, nanomicrosphere [10] technology has been developed as a new deep-control propulsion technology [11], with a controllable particle size [12], strong migration ability [13], good expansion performance [14], low cost [15], and good injection performance [16]. And other properties have achieved initial results in improving EOR, particularly in some low-permeability reservoirs. Microspheres and nanospheres also exhibit water absorption expansion [17] between particles through the “bridging effect” [18] to seal the neck of the formation hole. Nanospheres in field applications in Daqing [19], Shengli [20], Changqi [21], the Bohai Sea [22], and Xinjiang [23] have achieved better water control, oil stability, and improved oil recovery.

However, conventional nanospheres expand too quickly due to their microspheres [24], resulting in increased injection pressure and thus limiting their wider application. At the same time, due to the high water absorption rate [25], the nanoparticles swell before surface vegetation or oil pumping, which not only makes the water injection process complicated, but also makes it difficult to penetrate the oil reservoir for effective regulation and displacement [26].

Polymer microspheres [27,28] are regarded as cost-effective options for impeding water production in highly permeable formations by creating a physical barrier. Upon contact with reservoir water, the polymer microspheres, which possess strong hydrophilic groups, swell and can bridge or plug pore throats, thereby diverting subsequent water flows. They may undergo elastic deformation, allowing for migration and recapture. These microspheres can be tailored to match the characteristics of pore throats and are injectable into deeper reservoir sections. However, in formations that produce both water and hydrocarbons, polymer spheres struggle to target water production without impacting hydrocarbon output. High polymer solution viscosity can lead to low injectability. Moreover, polymer spheres exhibit poor resistance to high temperatures, high salinity, and shear, resulting in inadequate plugging strength and short-term consistency control. Therefore, Cheng [29] investigated the application of bilayer-coated microspheres (BCMS) in enhancing recovery and deep-sectioning performance in inhomogeneous reservoirs. Experiments and mathematical models showed that BCMS can effectively transport and plug porous media under high-temperature and high-salt conditions, reduce water runoff in high-permeability formations, and improve oil drive efficiency in low-permeability formations. They [30] also investigated the plugging performance and oil displacement efficiency of double-coated microspheres (BCMS) in heterogeneous reservoirs. The experimental results show that BCMS can effectively penetrate deep into the porous medium, increase the collision probability, form particle aggregates, effectively plug the high-permeability channels, and improve the oil recovery in the low-permeability region. The effects of the injection rate, particle concentration, slurry volume, and permeability ratio on the plugging performance of BCMS were also investigated. BCMS show good potential for application in improving reservoir productivity.

Therefore, the research and development of nanoparticles with slow-expansion properties has become an important research direction in the petroleum field. Given this requirement, a new type of nanoparticle design idea is proposed, which is to maintain no or slow expansion during the injection process and reach a certain multiple after reaching the target layer to realize the subsequent propulsion. Such particles must also be able to deform and move under a certain pressure difference to penetrate deep into the formation and then expand to achieve depth regulation and more effective propulsion. Based on the existing research base, we synthesized polymerizable amphiphilic crosslinkers and prepared polymer microspheres by the reverse microemulsion method. This method uses tiny droplets as the core of the chemical reaction and introduces two crosslinking agents with different stability. We investigated the influence of the total crosslinker, initiator, stirring speed, and reaction temperature on the preparation of the surface reaction method. In addition, the chemical structure, thermal stability, and micromorphology of the polymer microspheres were systematically characterized, and the slow-swelling and flooding properties were evaluated. The aim of this study is to provide new ideas and methods for the production of driven polymer microspheres. The dual-network structure of the microspheres is designed so that they can maintain their structural integrity during the expansion process while controlling the expansion rate through the decomposition of the unstable crosslinker, a controllability that is to some extent not available with conventional EOR methods.

The hydrophilicity of the crosslinker is increased by synthesizing a novel amphiphilic crosslinker (AE), a unique hydrophilicity that helps the nanomicrospheres to form a stable bi-network structure. We controlled the particle size of the microspheres at the nanometer level as much as possible by the reverse microemulsion method. By constructing networks of different stability of the nanomicrospheres, the first network is formed by a more stable crosslinking agent, which provides good strength and structural stability to the nanomicrospheres. The second network consists of less stable polymers that provide controlled conditions for the swelling of the nanomicrospheres. This structure allows the nanomicrospheres to maintain good structural integrity while achieving controlled swelling under the protection of a network with a high crosslink density.

## 2. Results and Discussion

### 2.1. Effect of Synthesis Conditions on Gently Expanded Nanosized Double-Mesh Microspheres

**Figure 1:**
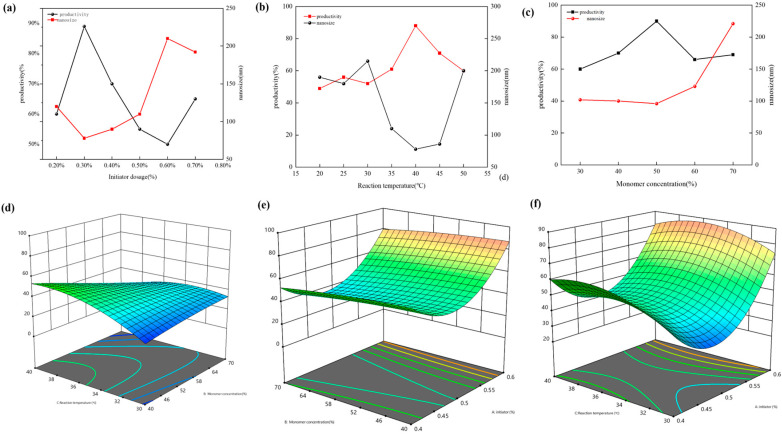
Effect of various factors on PAE yield and particle size Optimisation of synthesis conditions: (**a**,**d**) initiator dosage; (**b**,**e**) monomer concentration; (**c**,**f**) starting temperature.

We designed and optimized the synthesis conditions of double-mesh microspheres using Design-Expert 13 software with yield and particle size as indicators. The results are shown in Figure 1.

As shown in Figure 1a,b, when the proportion of the initiator dose was less than 0.3%, the polymerization reaction rate gradually accelerated with the increasing initiator dose, and the product gradually changed from cloudy to transparent. At the same time, the level of free radicals also increased. At this time, the chain growth rate was significantly higher than the chain termination rate, resulting in the growth of molecular chains. This led to the expansion of the three-dimensional network crosslinking structure of the nanomicrospheres and the corresponding increase in the initial particle size. Due to the increase in free radicals and reaction centers, the aggregation effect of the particles significantly exceeds that of nucleation polymerization. Therefore, we found that an initiator dose of 0.3% of the aqueous phase mass can effectively promote the reversed-phase microemulsion polymerization reaction.

From Figure 1c,d, we found that the polymerization process took longer with a lower monomer content. The temperature rise rate of microspheres is slow, and the size is large. As the monomer content is increased, the reaction becomes more violent and the temperature rises rapidly, thereby shortening the reaction time. Therefore, it is reasonable to synthesize double-cross-linked nanomicrospheres under the condition that the monomer content is 50%. At this time, the reaction process was relatively stable, and the product showed a transparent state and could produce a large amount of product after breaking the emulsion.

From Figure 1e,f, we can see that the yield increases and then decreases as the starting temperature increases, and the yield reaches about 90% when the temperature is 50 °C, and the yield decreases when the temperature is too high. This shows that the initiation temperature affects the activity of the initiator; when the temperature is low, the activity of the initiator is low, the release of free radicals is small, and the reaction is not complete. If the temperature is too high, free radicals are generated rapidly, the reaction is too violent, the chain termination time is advanced, the chain growth process is shortened, and the reaction is incomplete. When the initial temperature is lower than 50 °C, the temperature increases and the particle size of the product gradually decreases, which is due to the increase in the temperature being conducive to the release rate of free radicals. When the initial temperature is higher than 50 °C, the effect of the temperature increase on the particle size is not obvious, indicating that the free radical release can be accelerated when the initiation temperature is higher than 50 °C, prompting the reaction to proceed faster. Comparing the particle size and yield of the products under different initiation temperatures, it can be seen that the conversion rate of the polymerization reaction is higher at 50 °C, which is 89.8%, and the particle size is smaller, which is 88 nm. Therefore, 50 °C is taken as the optimal temperature for the polymerization reaction.

### 2.2. Effect of Overall Crosslinking on the Structure of Two Nanospheres

Based on the significant influence of the network density and slow-swelling properties of the dual-network structure of double-mesh particles, this study focuses on the effects of different addition ratios on the particle size in the microscopic morphology and slow-swelling properties of double-mesh nanospheres.

For the SEM observation and particle size analysis, different amounts of total crosslinking agent (MBA:AE = 1.1:1.05) were selected in the ratio of 0.6%, 0.8%, 1.0%, and 1.2%.

**Figure 2:**
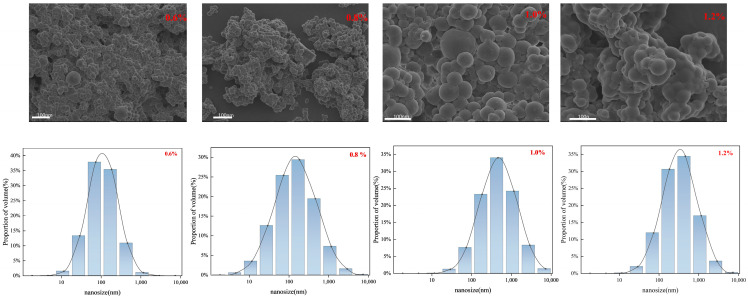
SEM of double-mesh microspheres with different amounts of crosslinking agent.

As shown in Figure 2, the particle size of the microspheres can be estimated from the particle size diagrams of 0.6% (75.45 nm), 0.8% (85.22 nm), 1.0% (110.30 nm), and 1.2% (150.34 nm) increases with the increasing crosslinking ratio, and we conclude that this is due to the amount of crosslinking agent added, the formation of moderately branched long polymer chains within the microspheres, and the dispersion of the amide group in the medium. Due to swelling, the amide group is hydrolyzed to carboxyl groups and electrostatic repulsion occurs between the adjacent carboxyl groups, stretching the long chains of the microsphere polymer and producing larger particle size microspheres. It can also be seen from the SEM that the crosslinking ratio is low, the microsphere has fewer internal crosslinking points, the formation of the three-dimensional network structure of the microsphere is incomplete, and the crosslinking agent content of the microsphere in the dry powder plays a crucial role in controlling the microparticle–spherical particle size. As the cross-linking ratio increases, the particle size of the microspheres gradually increases; as the cross-linking ratio continues to increase, ‘soft spheres’ with a complete network structure are formed.

### 2.3. Characterization of Double-Mesh Nanospheres

**Figure 3:**
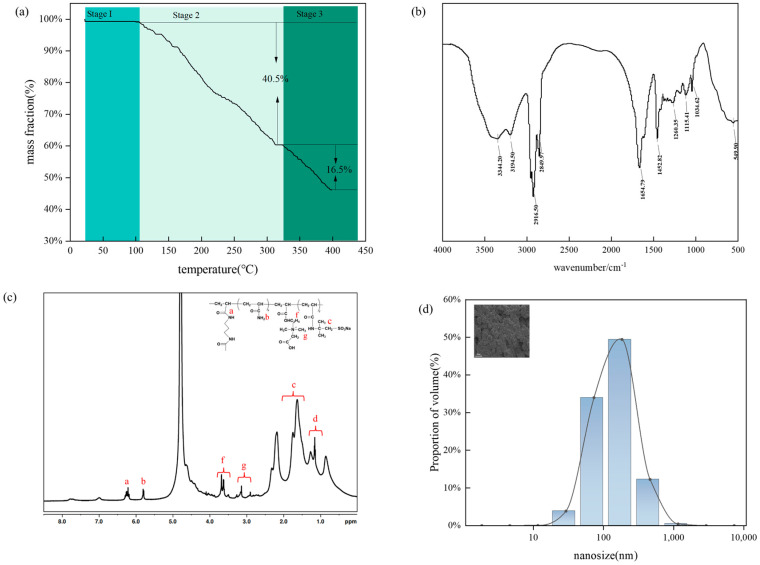
(**a**) Thermogravimetric (TGA) curve of microspheres, (**b**) infrared curve (FTIR), (**c**) hydrogen nuclear magnetic resonance (HNMR) spectrum of microspheres, (**d**) microsphere particle size.

The relevant structural and microscopic analyses of the double-mesh microspheres are shown in Figure 3.

Figure 3 shows (a) the thermogravimetric curve of double-mesh-type microspheres. The TGA curve can be divided into three stages: The first stage is 30 to 120 °C and is due to a loss of quality due to the evaporation of water. The freeze-drying treatment has lost more than 95% of the water, so there is no significant quality loss at this stage; in the second stage of 120–332 °C, this time, the water has completely evaporated, which is mainly due to the rupture of sulfonic acid radicals in the internal crosslinking point of the polymer gel due to the quality of the third-stage 332 loss. In the third stage, at a temperature of 332–440 °C, the main chain of microspheres begins to decompose and generates a lot of heat. The decomposition of the polymer microspheres is accelerated as the temperature increases at this stage. The reservoir temperature is usually in the range of 40–180 °C and the microspheres show good thermal stability in this temperature range.

As the FTIR spectrum of the microspheres in Figure 3b shows, the characteristic N-H bond stretching vibration peaks of the amide group are at 3344.20 and 3194.50 cm^−1^, the stretching vibration absorption peaks of the methylene group are at 2916.50 and 2849.97 cm^−1^, and the strong absorption peak is at 1654.79. The strong absorption peak at cm^−1^ is the C==O characteristic stretching vibration absorption peak of the amide group, the deformation vibration absorption peak of the methylene group at 1452.82 cm^−1^, the stretching vibration peak of the ester bond C-O-C at 1115.41 cm^−1^, and the absorption peak at 1034.62 cm^−1^ are the homemade laboratory crosslinker AE in the -R3N+ absorption peak. There is no stretching vibration absorption peak of c = c in the range of 1660 to 1690 cm^−1^, indicating that the reaction of each reactive monomer is sufficiently proven that AM copolymerizes with MBA and AE. This indicates that the microspheres were successfully prepared, and the expected double-network structure of the microspheres was realized.

The hydrogen NMR-^1^HNMR spectrum of the microspheres is shown in Figure 3c. All signals appearing in the spectrum were assigned to the corresponding protons. The copolymerization rates of the copolymers were calculated by ^1^HNMR spectrum analysis. The chemical shifts of the H atoms at point a were in the range of 6.50 to 7.00 ppm, at point b, in the range of 5.55 to 6.01 ppm, and at points f and g, in the range of 3.00 to 3.90 ppm. From the structure of the copolymer microspheres, it can be seen that nanomicrospheres containing polymerizable amphiphilic crosslinkers can also be successfully synthesized and that there is a connection between the structures and the microspheres. From Figure 3d, it can be seen that it is mainly concentrated in the range of 56–98 nm, with an average particle size of 86 nm. In addition, the particle size distribution of the microspheres is also in the range of 56–98 nm, as observed by scanning electron microscopy (SEM), which is consistent with the test results of the laser particle sizer. The SEM images also show this. The boundaries of these particles are clearly defined and the dispersibility is good. This indicates that the nanomicrospheres have good sphericity and dispersibility.

### 2.4. Slow-Swelling Properties of Double-Mesh Microspheres

The slow-swelling performance of PAE was better than that of PAM microspheres.

**Figure 4:**
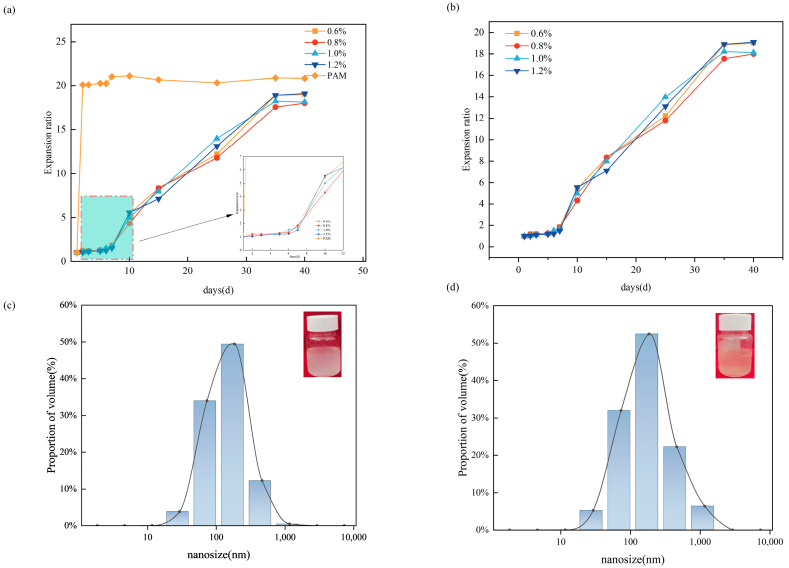
Slow-expansion performance of microspheres (**a**) for comparison with single-mesh microspheres; (**b**) for the slow-expansion time of microspheres with different crosslinker additives; (**c**) initial nanoscale microsphere size; (**d**) slow expansion at 80 °C for 48 h.

From Figure 4a,b, we can see that the swelling rates of PAE and PAM first show a slow and then fast trend and finally stabilize, and PAM reaches the swelling equilibrium after 5 days. PAE reaches equilibrium after 45 days with a swelling factor of about 16. This phenomenon is attributed to the fact that the stabilizing crosslinker and the unstabilizing crosslinker work together to increase the crosslinking density, thereby forming a double-network microsphere structure. In this structure, water molecules must penetrate both networks to reach the interior of the microspheres. This process increases the difficulty for water molecules to penetrate the microspheres, effectively extending the time it takes for water molecules to penetrate the microspheres. The regulation of the water absorption and swelling rate of the microspheres was realized, thereby slowing down the water absorption and swelling process. From Figure 4c,d, it can be seen that the double-mesh-type-based nanoparticles have significant retarded properties in the swelling process. The nanoparticles show different slow-swelling times at different temperatures. The reason for this lies in the different degradation rates of the unstable crosslinker AE under different temperature conditions.

### 2.5. Analysis of Synthesis Mechanism and Slow-Swelling Mechanism of Microspheres

**Figure 5:**
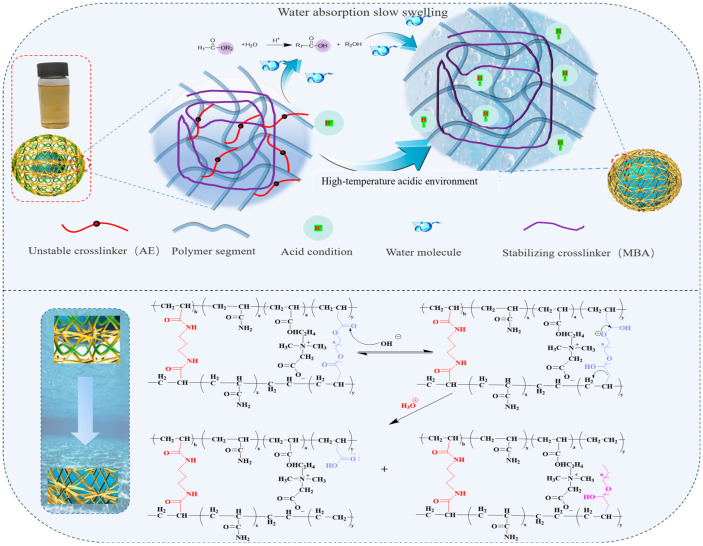
Mechanism of slow expansion of bi-crosslinked nanomicrospheres.

The double-network-type microspheres with their double-network mechanism are shown in Figure 5. Two crosslinkers with different stability under the APS-SHS redox system polymerize with the monomers to produce doubly cross-linked nanomicrospheres. The chains formed by the stabilizing crosslinkers serve as the first network and the polymerizable amphiphilic crosslinker serves as the second network. Regulation of the dual-network density of the microspheres is achieved by adjusting the molar concentration of the second network to that of the first network; the nanomicrospheres are tightly wrapped by the first network and then loosely cross-linked by the second network to achieve a highly asymmetric microsphere structure. Because it is a kind of dual-network-type nanomicrosphere, the first network with a high crosslinking density has a high elastic modulus, which allows the microspheres to maintain good strength and structure.

On the contrary, the second network with a loose crosslinking density provides controlled conditions for the expansion rate of the nanomicrospheres. The swelling mechanism of microspheres mainly includes hydrogen bond formation, hydrolysis, and osmotic pressure-controlled diffusion processes. When the microspheres come into contact with water molecules, the water molecules penetrate the network structure of the microspheres and interact with the hydrophilic groups contained therein, resulting in the formation of hydrogen bonds and increasing the affinity between the microspheres and the water molecules. In addition, the spatial network structure gives the microspheres viscoelastic properties, and by absorbing water molecules, the molecular chains are in a stretched state in irregular curls, creating a strong cohesive force. When these forces reach dynamic equilibrium, the microspheres complete the swelling process and reach the saturated water state. The swelling mechanism of microspheres can be designed for the slow-swelling mechanism of dual-network microspheres as shown in Figure 5, the core mechanism of which is to control the swelling rate of microspheres by controlling the decomposition of the unstable crosslinker. The swelling process of microspheres is not only limited by the water absorption and swelling ability of the microspheres themselves but also influenced by the decomposition of the unstable crosslinker. The decomposition of the unstable crosslinker creates a large number of loose network structures around the microsphere particles.

Due to the difference in osmotic pressure, the high-salt solution can easily penetrate into the internal structure of the microspheres and further promote their swelling. When the unstable crosslinker undergoes thermal decomposition, in contrast to the undecomposed stabilized crosslinker, a loose structure similar to a mushroom cloud is formed, which maintains a high crosslinking density, thus ensuring the structural integrity and density of the microspheres. In summary, the slow-swelling process of doubly cross-linked microspheres is a complex physicochemical process involving hydrogen bond formation, hydrolysis, osmotic pressure-driven diffusion, and decomposition of the unstable crosslinker. The synergistic effect of these mechanisms allows the microspheres to achieve swelling while maintaining structural integrity and compaction.

### 2.6. Analysis of Interfacial Tension and Drive Modulation Properties of Double-Mesh Microspheres

**Figure 6:**
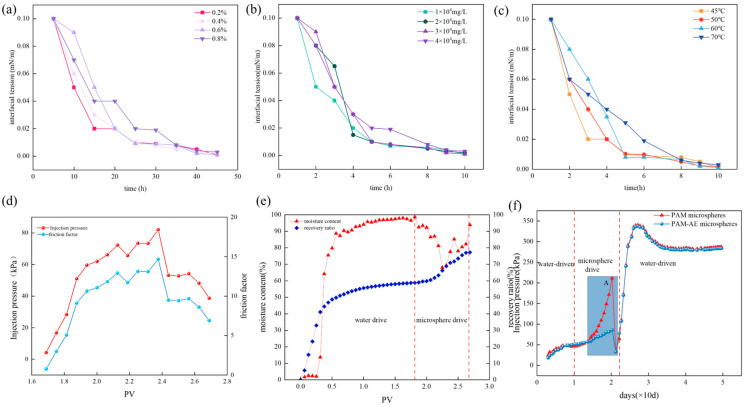
(**a**) For different concentrations; (**b**) for different mineralizations; (**c**) for the interfacial tension of the microspheres at different temperatures; (**d**) for the injection pressure and the resistance coefficients of the microspheres; (**e**) for PAM vs. PAE microsphere pressure; (**f**) for the microsphere drive modulation performance.

From Figure 6a, it can be seen that the interfacial tension decreases faster as the concentration of the aqueous solution of the microspheres increases, which proves that the microspheres have lower interfacial tension properties. Furthermore, in Figure 6c, the variation in interfacial tension at different temperatures was tested in a microsphere solution with a concentration of 0.7%. The experimental results show that the time required to achieve low interfacial tension increases with the increasing temperature. However, the final interfacial tension could stabilize below 1.0 × 10^−3^ mN/m regardless of the temperature change, indicating that high temperatures have no significant influence on the interfacial tension value of the microspheres. Figure 6b shows the effect of different degrees of mineralization on the interfacial tension of microsphere solutions with a concentration of 1%. The experimental results show that the decrease in the interfacial tension value of the microspheres takes longer as the degree of mineralization increases. However, the interfacial tension values of the nanomicrospheres were always able to remain below 1.0 × 10^−3^ mN/m during the test period within 10 h, which indicates that the degree of mineralization also has only a small influence on the interfacial tension of the microspheres. This indicates that the microspheres have low interfacial tension under reservoir conditions, which helps to improve the oil repulsion efficiency of the reservoir.

In summary, the nanomicrospheres exhibited good interfacial tension properties, and their interfacial tension values were less affected by the concentration, temperature, and mineralization. It can be seen from Figure 6d that the injection pressure was kept stable at 0.03 MPa after water propulsion. However, during the injection of microspheres, the injection pressure experienced significant fluctuations, showing a rising and falling trend, with a fluctuation range of 0.03 to 0.43 MPa, and the drag coefficient was significantly increased by 13.3 times. During subsequent water trips, the pressure continued to exhibit fluctuating changes characterized by a range of 0.43 to 0.24 MPa, a pressure level significantly higher than the stabilized pressure at the end of a single water trip, and the final drag coefficient stabilized at about 8.

As depicted in Figure 6d–f, the new microspheres PAE exhibit excellent slow swelling characteristics compared to the conventional polymer microspheres (PAM). At point A, the PAM microspheres reach a maximum breakthrough pressure that is much higher than the breakthrough pressure of the PAE microspheres. At 60 °C, PAE microspheres expand 16 times faster, with a slow expansion of 45 days. In addition, the water plugging rate of PAE reaches 98.3%, the oil repulsion rate is 73.11%, and the recovery can be improved by 11.23%. These data strongly demonstrate the potential of the new type of microspheres in improving oilfield recovery.

## 3. Experimental Section

### 3.1. Materials and Instruments

#### 3.1.1. Materials

Dimethyl aminoethyl methacrylate (DM), acrylamide (AM), and ammonium persulfate (APS) were from Shanghai Macklin Biochemical Technology Co., Ltd. (Shanghai, China).sodium bisulfite (SHS), phenothiazine, and sodium chloroacetate were bought from Shanghai Aladdin Biochemical Technology Co., Ltd. (Shanghai, China). N,N-methylene bisacrylamide (MBA), was from Tianjin Zhonglian Chemical Reagent Co., Ltd. (Tianjin, China). 2-acrylamide-2-methylpropanesulfonic acid (AMPS), was bought from Wuxi Sandry Technology Development Co., Ltd. (Wuxi, China).

#### 3.1.2. Experimental Equipment

Experimental equipment: SP-20002 electronic balance SP-20002 Haining City Shengbo Weighing Instrument Co., Ltd. (Haining, China).; constant temperature bath box (laboratory homemade); KX-1613QTD ultrasonic oscillator Beijing Kexi Co. (Beijing, China). NKT-N9 Nano Laser Particle Size Analyzer, Shandong Nexter Analytical Instrument Co., Ltd. (Jinan, China).; NicoletIS50 FTIR Spectrometer, Thermo Fisher Scientific (China) Ltd. (Shanghai, China).; EOL JSM-7800F Field Emission Scanning Electron Microscope, Nippon Electron Co. (Shanghai, China).

### 3.2. Methods

#### 3.2.1. Preparation of a Polymeric, Amphiphilic Crosslinker

**Figure 7:**
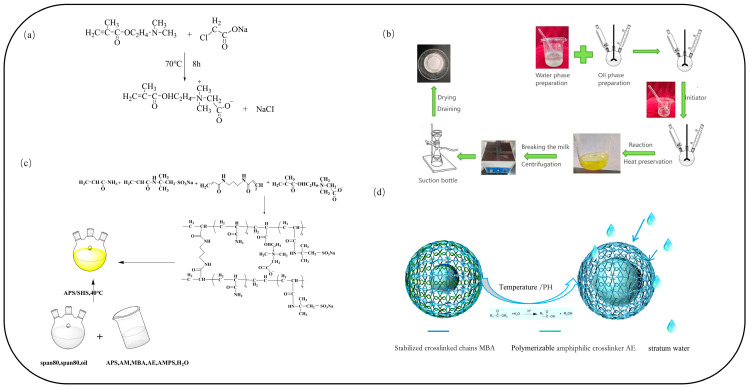
(**a**) Modification of AE crosslinking agent; (**b**) flow chart for the preparation of double-mesh nanospheres by reverse microemulsion method; (**c**) synthesis of double-mesh nanospheres by reverse microemulsion method; (**d**) retarded expansion of double-mesh microspheres.

As shown in Figure 7a, for the synthetic polymerizable amphiphilic copulative agent, add 5.5 g of dimethylamine ethyl ester (DM) and a small amount of phenothiazine to the three-necked condensing reflux flask, increase the temperature to 60 °C, add 4.2 g of sodium chloroacetate, and heat to 70 °C. After 7 h of reaction, allow the reaction liquid to cool, and calculate the yield and content.

#### 3.2.2. Preparation of the Polymer Microsphere PAE

Double-networked nanomicrospheres were prepared as depicted in Figure 7c. This process involved weighing 6.56 g of Span 80 and 2.19 g of Tween 80, which were then added to 23 g of white oil and thoroughly mixed to form the oil phase. Next, 4.8 g of acrylamide, 0.3 g of stabilizing crosslinker (MBA), 2.3 g of 2-acrylamide-2-methylpropanesulfonic acid, and 0.5 g of amphiphilic crosslinking agent (AE) were weighed to create an aqueous solution of the monomer with a mass fraction of 50%. The pH of the aqueous phase was adjusted to 6–8 using a 10% NaOH solution. The aqueous phase was slowly added to the oil phase with continuous stirring until a translucent microemulsion was formed. Polymerization was initiated by adding 0.3% APS/SHS after nitrogen purging for 30 min. Following the initial warming phase, the reaction was maintained for an additional 2 h to obtain the microemulsion. The emulsion was then broken with anhydrous ethanol, washed repeatedly, and the particles were precipitated and filtered under reduced pressure. Finally, the double-networked nanomicrosphere particles were obtained as a powder by drying at 80 °C in an oven for 12 h.

#### 3.2.3. Preparation of the PAM for the Polymer Microspheres

The conventional polymer microspheres (PAM) were prepared by reverse-phase suspension. Amounts of 42 g of white oil and 7.7 g of Tween 80 were weighed and mixed evenly in a 250 mL three-necked flask. For the oil phase, N2 was vented for 10 min. The 38.6 g of deionized water, 9 g of AM, and 1 g of AMPS were dissolved, and the pH of the system was adjusted to 7–8 with a 20% NaOH solution, then 0.5 mL of 2% MBA with stirring aqueous solution was added. The aqueous solution was slowly added to the three-necked flask using a constant-pressure drop funnel, the mixer was opened and stirred at 600 rpm for 1 h, heated to 40 °C, and 0.5 mL of 3% mass fraction APS and 0.4 mL SHS at a mass fraction of 1% were added, reacted at 300 rpm for 3 h, and the milky emulsion product was PAM.

### 3.3. Structural Characterization and Performance Tests

FTIR: the surface functional groups of P (AM-AE) were analyzed using the IS 50 Fourier-transform infrared spectrometer with a wavelength range of 4000 to 400 cm^−1^.

TGA: the P (AM-AE) thermal stability was characterized after drying treatment (12 h) at N_2_ from room temperature to 450 °C under atmosphere.

SEM: Micromorphology of P (AM-AE) by scanning electron microscope. Particle size analysis: the particle size distribution of P (AM-AE) was characterized using a laser particle sizer.

### 3.4. Water Absorption and Expansion Performance

m0P (AM-AE) and PAM microsphere powder were loaded into a 400-purpose sieve mesh, placed in a beaker, and placed in a 60 °C incubator. The sample was taken regularly; after absorbing the surface moisture with water absorption paper, the quality of the sample was checked after water absorption, recorded as m1, and the expansion doubling speed was m0/m1.

### 3.5. Physical Transfer Performance

The displacement performance of the double-mesh microsphere system in the reservoir was mainly evaluated by the resistance coefficient, plugging rate, and recovery efficiency. The experimental steps were as follows:

(1) The sand filling pipe was filled, the dry weight of the sand filling pipe was weighed, saturated simulated water was injected, the wet weight of the sand filling pipe was weighed, and the porosity of the sand filling pipe and the pressure difference P were determined; when the pressure increases were stable, the permeability of the sand filling pipe was calculated according to the Darcy formula.

(2) The dehydrated crude oil was injected into the sand filling pipe, the volume of water drained from the collector, namely the saturated crude oil volume, was recorded and put into a 90 °C oven for 24 h.

(3) Formation simulation water was injected into the sand filling pipe until the water content at the outlet end was more than 98% to calculate the recovery rate currently.

(4) After a mass concentration of 5000 mg/L (0.15%) was injected into the pipe, the simulated formation water 1 PV and the microsphere suspension 1 PV at an injection rate of 0.8 mL/min were added at both ends. The tube containing the displacement fluid was sealed and placed in a constant-temperature incubator at 60 °C for 30 days. Then, the simulated formation water was injected at a flow rate of 0.5 mL/min to record the change in the pressure gauge with the injection amount. The water composition of the simulated formation is shown in Table 1.

(5) The water content of the secondary water driven to the outlet end of the sand filling pipe was greater than 98%, and the pressure difference *P* was recorded as the permeability of the sand filling pipe (*K_b_*). After final recovery, the resistance coefficient was recorded according to the following formula (F_R_) and the clogging rate (η) calculated.

(1) $$F_{r}=\frac{P_{2}}{P_{1}}$$

(2) $$F_{r}=\frac{P_{1}}{P_{0}}$$

(3) $$\mu =\frac{k_{a}-k_{b}}{K_{a}}\times 100\%$$

In the above equation, *F_r_* is the resistance coefficient; *P*_0_ is the stable pressure of the water-measured sand-filled pipe, kPa; *P*_1_ and *P*_2_ are the stable pressures before and after the injection of microspheres, kPa, respectively; is the sealing efficiency of the moderating agent, %; and *K_a_* and *K_b_* are the permeability of the primary and secondary water drives, μm^2^, respectively.

## 4. Conclusions

(1) Slowly expanding dualnetwork nanomicrospheres with an average particle size of 76 nm were prepared under the optimal preparation conditions of an oil phase to water phase mass ratio of 3:2, a stirring speed of 550 rpm, and an unstable crosslinking drug dose of 0.80%, a total crosslinker dose of 0.6% (based on the total mass of monomers), an initiator dose of 0.30%, and a reaction temperature of 45 °C.

(2) Infrared spectral analysis shows that we can verify whether the monomers have been successfully polymerized and the microspheres are well synthesized. Scanning electron microscopy (SEM) showed that the surface network structure of the microspheres was denser, and the delayed expansion performance was improved at different crosslinker dosages. Under different temperatures and mineralization conditions, the expansion rate of the slow-expanding microspheres was 18.2 times in 35 days, which showed excellent slow-expansion performance and could maintain the original expansion degree.

(3) The mechanism of slow expansion of slow-expanding double-network microspheres lies in the internal construction of two net-like structures with significant stability differences. By controlling the amount of crosslinking agent, the stabilization time of these two networks can be controlled and thus the expansion time of the microspheres can be regulated. This mechanism ensures that the microspheres can expand gradually and stably according to the specified requirements in a certain environment.

(4) The results of the interfacial tension experiments show that the final interfacial tension of the microspheres at different temperatures is 1.0 × 10^−3^ mN/m, indicating that the nanomicrospheres have excellent interfacial tension properties and their interfacial tension values are less affected by the concentration, temperature, and mineralization degree. The sand-filled tube driving experiment shows that the recovery rate with microsphere driving is 72.2%, which improves the recovery rate by 11.25%, proving that the microspheres have excellent modulation driving performance.

## Figures and Tables

**Table 1:** Simulated formation water–ion ratio.

Ion Type(mg/L)	Na^+^	Ca^2+^	CI^−^	Total
Concentration	18.48 × 10^3^	1.08 × 10^3^	30.45 × 10^3^	5 × 10^4^

## Data Availability

Data are contained within the article.
